# Catheter ablation for failed surgical maze: comparison of cut and sew vs. non-cut and sew maze

**DOI:** 10.1007/s10840-019-00509-y

**Published:** 2019-01-31

**Authors:** Roger A. Winkle, William Fleming, R. Hardwin Mead, Gregory Engel, Melissa H. Kong, Jonathan Salcedo, Rob A. Patrawala, Luis Castro, Vincent Gaudiani

**Affiliations:** 1 Silicon Valley Cardiology, Palo Alto Medical Foundation and Sutter Health, 1950 University Avenue, Suite 160, E, Palo Alto, CA 94303 USA; 2 Sequoia Hospital Heart and Vascular Institute, Redwood City, CA USA; 3 Pacific Coast Cardiac & Vascular Surgeons, Redwood City, CA USA

**Keywords:** Atrial fibrillation, Atrial flutter, Maze operation, Ablation

## Abstract

**Purpose:**

To compare findings in patients undergoing atrial fibrillation(AF) and/or atrial flutter(AFl) ablation after failed cut and sew (CS) vs. non-cut and sew (NCS) surgical maze.

**Methods:**

We compared 10 patients with prior CS to 25 with prior NCS maze undergoing catheter ablation after failed maze.

**Results:**

Patient demographics: Age 68.3 ± 8.7 CS vs. 68.2 ± 9.2 NCS(*P* = 0.977), male 70% CS vs. 72% NCS(*P* = 1.000), LA size 5.11 ± 0.60 cm CS vs. 4.54 ± 0.92 cm NCS(*P* = 0.096), sternotomy 100% CS vs. 64% of NCS(*P* = 0.036). Concomitant heart surgery in 100% CS and 68% NCS(*P* = 0.073). NCS used radiofrequency 84%, cryoablation 8%, microwave 4%, and ultrasound 4%. All maze operations targeted pulmonary vein (PV) isolation. The maze also targeted the mitral isthmus 100% CS vs. 36% NCS(*P* = 0.001) and the tricuspid isthmus 90% CS vs. 40% NCS (*P* = 0.018). Maze failure arrhythmia mechanism was AF 0% CS and 56% NCS (*P* = 0.0006). Nine CS pts failed for AFl and 1 for RA tachycardia. For NCS pts, 11 failed for AFl. CS isolated 94% of PVs and NCS isolated only 26% of PVs (*P* < 0.0005). At EPS, clinical and induced arrhythmias were ablated and non-isolated PVs were isolated. After final ablation, arrhythmia-free rates were 60% for CS and 52% for NCS (*P* = 0.723) after 2.99 ± 2.35 years.

**Conclusions:**

After failed surgical maze, CS isolated nearly all PVs and NCS never isolated all PVs and the clinical rhythm was more frequently AF for NCS and AFl for CS. CS remains the surgical gold standard for durable PV isolation.

## Introduction

James L Cox introduced the first surgical procedure for atrial fibrillation (AF) and the original Cox maze procedure has undergone several iterations [1]. The first and second iterations were discontinued because of chronotropic insufficiency, a higher rate of post-surgical pacemaker implantation, occasional left atrial (LA) dysfunction, and the difficulty of portions of the surgery [2]. The Cox maze III procedure achieved wide popularity as both a stand-alone procedure and as an add-on procedure for patients undergoing other types of open-heart surgery [3]. The Cox maze III procedure involved a box lesion consisting of a through and through surgical incision around all 4 PVs (PVs). In addition to isolating the veins, this box also isolated the posterior wall. The Cox maze III also involved a mitral isthmus line, a tricuspid isthmus line, a line from the superior vena cava to the inferior vena cava, and excision of the LA appendage. With newer ablation tools such as bipolar radiofrequency ablation, the Cox maze IV procedure evolved and many of the surgical incisions were replaced by radiofrequency or cryoablation lesions [4]. In the original Cox maze IV, the right and left PVs were encircled as two circles and an inferior line leaving most of the posterior LA electrically connected to the remainder of the atrium. Later, a superior ablation line was added for full posterior wall isolation [5]. Variations of the Cox maze IV procedure have been done at the time of open-heart surgery via sternotomy or as stand-alone procedures through mini thoracotomies or ports, both as on-pump or off-pump procedures. Procedures have used focused ultrasound [6], and surgical and catheter-based ablations have been combined for a “hybrid” procedure [7]. Many times, these surgical procedures eliminate atrial arrhythmias. However, some patients experience arrhythmia recurrence and come for catheter ablation to complete the goal of arrhythmia elimination. In this study, we examined patients undergoing catheter ablation after a failed surgical maze procedure. We compared the patients who had undergone a classic cut and sew (CS) maze to those who had undergone a non-cut and sew (NCS) maze.

## Methods

### Patient population

The subjects were patients at Sequoia Hospital, Redwood City, California undergoing catheter ablation for symptomatic recurrent atrial arrhythmias after a failed surgical maze operation. All signed written informed consent. The study was approved by the Western Institutional Review Board.

### Clinical data collection

All patients were part of the prospectively collected Sequoia Hospital AF ablation database. For each patient, we recorded age, gender, duration of AF or atrial flutter(AFl), time from prior surgical maze operation to first catheter ablation, prior antiarrhythmic drug therapy, CHADS_2_ score, prior cardioversions, body mass index, LA, size and the presence of prior strokes/TIA’s, hypertension, coronary artery disease, and dilated cardiomyopathy. We retrospectively reviewed all electrophysiologic findings for the catheter ablation procedures.

### Surgical data collection

We retrospectively obtained the prior surgical operative notes for all the maze surgical procedures. For each operative maze, we determined the type of ablative energy or surgical incisions and the lesion set performed. The NCS operations were done by a variety of surgeons and they did not follow a single protocol. The CS procedures were all performed by two of the authors (VG and LC). The technique used was very similar to the original Cox maze III [3] with a few differences to facilitate the operation. The operation followed the objectives for that operation, namely, complete pulmonary vein (PV) isolation and division of major macroreentrant circuits in both atria. PV isolation was achieved by sharply dividing the PV cuff from the rest of the LA. A 2-cm tissue isthmus of intact atrium was left near the LA appendage to facilitate closure, and this isthmus was treated with linear cryoablation lesions across the isthmus on both the endocardial and epicardial surfaces. All cryoablation lesions were at − 60 °C for 2 min on each side of the tissue. Three potential macroreentrant circuits were divided in the right atrium. A full thickness line was cut with cautery from the lateral tricuspid annulus to the inferior vena cava to prevent a caval-to-caval loop. A second line was cut from the medial tricuspid annulus up into the right atrial (RA) appendage to interdict a loop around the tricuspid annulus or the RA appendage. A vertical right atriotomy and a standard left atriotomy were used to facilitate placing a linear cryoprobe lesions in the LA and another in the right atrium that were parallel and trapped a piece of atrial septum to interrupt a loop around the fossa ovalis. The LA appendage was always excised. A cryoablation line was placed from the PV encircling incision to the mitral annulus on the endocardial surface, and the line was repeated on the epicardial surface including the coronary sinus to preclude a loop around the mitral valve or through the coronary sinus. This procedure took about 40 min of clamp time.

### Ablation protocol

Our ablation protocol has been described previously [8]. Antiarrhythmic drugs were stopped at least 5 half-lives and amiodarone at least 3 months before ablation. General anesthesia was used in all and venous access was from the right groin only. A 9F Boston Scientific (Natick, MA) Ultra Ice™ catheter guided the transseptal puncture, done using a standard needle or a 71-cm Baylis (Montreal, QC) NRG™ radiofrequency needle. The mapping and ablation catheters were both placed in the LA across a single transseptal puncture using a standard SL1 transseptal sheath or a St. Jude Agilis steerable sheath. Ablations were done using an open-irrigated tip ablation catheter at 50 W for short durations [9]. Patients had a femoral or radial arterial line.

### Mapping protocol

3D geometry and mapping were done using the St. Jude EnSite Velocity system with Precision Mapping Module or a Biosense Webster Carto mapping system. A 7F 20 electrode catheter (St. Jude Livewire™) was placed around the tricuspid valve annulus with the distal poles in the coronary sinus. Following access to the left atrium (LA), a 3D geometry was created. Patients who were in their clinical atrial arrhythmia at the start of the case had activation mapping undertaken prior to PV isolation, when needed. A circular mapping catheter (St. Jude Reflexion Spiral™) with 20 electrodes and a 15–25-mm variable loop or a Biosense Webster Lasso was used as a roving mapping catheter. Mapping points not near the static model geometry surface were excluded from analysis. Both color isochronal and propagation maps were generated. The circuit of each macroreentrant atrial flutter was determined as previously described [10], and entrainment was used when necessary. Microreentrant atrial flutter or focal atrial tachycardia was defined as an arrhythmia where the origin of the arrhythmia occurred in a very small area (< 1 cm) and radiated outward from that site. Arrhythmia induction with and without isoproterenol was undertaken until all inducible organized atrial arrhythmias had been mapped and ablated. All PVs were checked to determine if they were isolated with both entrance and exit block and any which were still connected were re-ablated to complete PV isolation.

### Follow-up

Some patients were treated with antiarrhythmic drugs and/or cardioverted during the 3 months following the ablation. If given, antiarrhythmic drugs were stopped after a 3-month blanking period. Patients sent daily transtelephonic ECG strips for 1–3 months after ablation and were seen at 3 and 12 months at which time 7–14 days of continuous ECG monitoring was done. Thereafter, patients were seen directly or contacted by phone at least annually and arrhythmia records obtained from hospitals and referring physicians. ECG recorders were reissued for any arrhythmia symptoms. Pacemaker AF data were utilized when available. Any repeat ablations were done after a 3-month blanking period. A successful ablation was defined as no AF, atrial flutter, or tachycardia lasting more than 30 s off all antiarrhythmic drugs after a 3-month blanking period.

### Data analysis

Continuous data were described as mean + standard deviation and counts and percents if categorical. Group comparisons between CS and NCS were done with chi-squared analysis or Student’s *t* tests. Freedom from all atrial arrhythmias off antiarrhythmic drugs was determined at last follow up. Statistics were done using XLStat, 2015 (Paris, France). All statistical tests were two tailed with a *P* value of < 0.05 considered as statistically significant.

## Results

### Patient population

The patient population consisted of 35 patients undergoing surgical maze procedure with clinical failure due to recurrence of symptomatic atrial arrhythmias post-surgical maze. They underwent subsequent radiofrequency catheter ablation from 2006 to 2015. Ten patients underwent a traditional modified Cox III CS surgical maze procedure and 25 underwent NCS surgical procedures. Table 1 compares the clinical characteristics of the CS versus NCS patient groups. There were no differences between the two groups regarding age, gender, BMI, and duration of AF. The NCS patients had more diabetes and hypertension as well as higher CHADS2 scores. Although the LA dimension prior to catheter ablation was larger in the CS group, this did not reach statistical significance.

**Table 1:** Comparison of clinical characteristics of CS and NCS maze patients

	Cut and sew	Non-cut and sew	*P* value
Number of patients	10	25	n/a
Age(years)	68.3 + 8.7	68.2 + 9.2	0.977
Male gender	70%	72%	1.000
Duration of AF (years)	7.7 + 9.5	9.0 + 6.8	0.652
No. of drugs failed	1.30 + 1.05	1.25 + 1.05	0.283
LA size (cm)	5.11 + 0.60	4.54 + 0.92	0.096
Average CHADS_2_ score	0.88 + 0.97	1.49 + 1.14	<0.0005*
Hypertension	46.7%	61.0%	<0.0005*
Diabetes	8.9%	14.4%	<0.0005*
Body Mass Index	28.9 + 4.8	27.3 + 6.0	0.4583
Prior cardioversion	46.9%	47.2%	0.914
Prior stroke/TIA	6.9%	8.0%	0.399
Time from maze to catheter ablation	2.83 + 1.92	4.84 + 3.59	0.1049

### Surgical maze procedure

A sternotomy incision was used in 100% of patients with a CS maze versus 64% with a NCS maze (*P* = 0.036). The remainder of the NCS maze operations were done via bilateral thoracotomies or ports. PV isolation was the target of ablation for all CS and NCS maze procedures. For the NCS procedures, the modality used was radiofrequency 84%, cryoablation 8%, microwave 4%, and ultrasound 4%. The surgical maze targeted the mitral isthmus in 100% of patients in the CS group versus 36% for the NCS group (*P* = 0.001). For the NCS operations, the modality for mitral isthmus ablation was the same as used to create PV isolation. The tricuspid isthmus was targeted in 90% of CS procedures using an incision from the tricuspid valve to the inferior vena cava versus 40% who had this line created in the NCS group (*P* = 0.018). One of 10 (10%) patients in the CS group and 4 of 25 (16%) of patients in the NCS group also had a superior vena cava to inferior vena cava line created.

### Mechanism of clinical arrhythmia failure after surgical maze

The arrhythmia resulting in failure of the maze procedures is shown in Table 2. The failure arrhythmia was AF in 0% of CS patients and 56% of NCS patients (*P* = 0.0006). Nine CS patients failed for atrial flutter and 1 for RA tachycardia. For the CS patients, 7 of the 9 atrial flutters involved either the mitral or tricuspid isthmus. For the NCS patients, 11 failed for atrial flutter which was a complex LA flutter in 5, a mitral isthmus flutter in 3, and a tricuspid isthmus flutter in 3. At EP study, 6 “non-clinical” arrhythmias were induced in 4 CS patients and 17 non-clinical arrhythmias were induced in 7 non-CS patients.

**Table 2:** Arrhythmia resulting in failure of the surgical maze operations

	Cut and sew	Non-cut and sew	*P* value
Atrial fibrillation	0(0%)	14(56%)	0.005
Complex LA flutter	2(20%)	5(20%)	1.00
Mitral isthmus LA flutter	3(30%)	3(12%)	0.322
RA isthmus flutter	4(40%)*	3(12%)	0.155
RA tachycardia	1(10%)	0(0%)	0.286

### Pulmonary vein isolation documented at catheter ablation

**Fig. 1:**
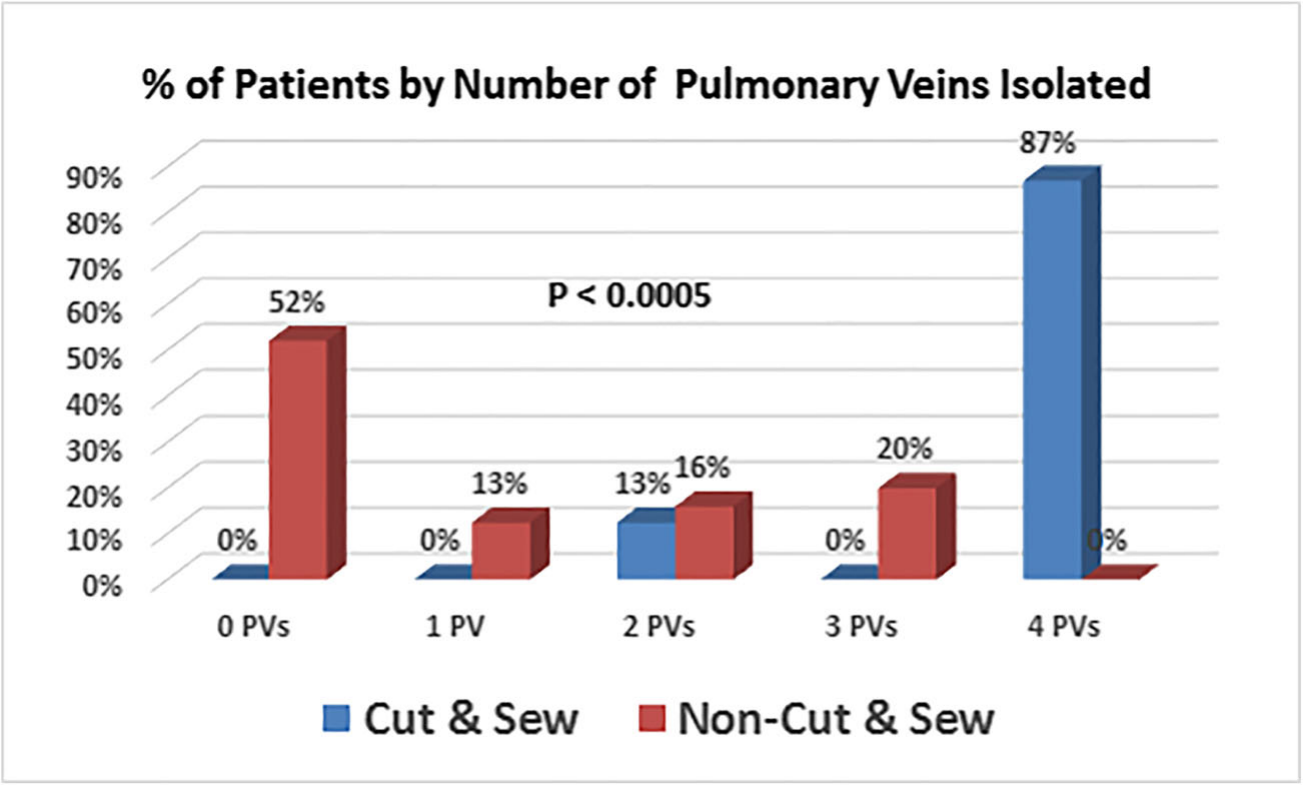
The percent of patients and the number of PVs isolated for the cut and sew and NCS operations. It can be seen the CS operation resulted in 87% of patients with all 4 veins isolated vs. 0% for the NCS patients. The NCS operation failed to isolate even a single vein in 52% of patients

Two CS patients with RA flutter and no other inducible arrhythmias only underwent a right-sided procedure. The status of the PVs was evaluated for all 33 remaining patients. Thirty-two patients had the usual 4 PVs and 1 NCS patient had a fifth vein. For the CS patients, 30 of 32 veins (94%) were isolated by their surgical procedure. The two non-isolated veins occurred in the same patient so that 90% of CS patients had all PVs isolated by their surgical procedure. For the NCS patients, only 26 of 101 veins were isolated (26%) (*P* < 0.0005 compared to CS patients). Thirteen patients (52%) had no PVs isolated, 3 (12.5%) had 1 vein isolated, 4 (16%) had 2 veins isolated, 5 (20%) had 3 veins isolated, and no patient had all veins isolated (Fig. 1). The vein isolation rates for the various PVs were right upper PV 31%, right inferior PV 32%, right middle PV(*n* = 1) 0%, left upper PV 24%, and left inferior PV 20%.

### Concomitant procedures

At the time of their surgical maze, many patients underwent concomitant surgical procedures including aortic valve replacements, mitral valve repairs, mitral valve replacements, coronary bypass operations, and combinations of these operations. All 10 (100%) of CS patients underwent concomitant heart surgery whereas 17 (68%) of NCS patients underwent concomitant surgery (*P* = 0.073).

### Radiofrequency ablation

The time from the surgical maze procedure to the initial catheter-based ablation was 4.27 + 3.26 years. This was 2.8 + 1.9 years for the CS patients and 4.8 + 3.5 years for the NCS patients. All patients underwent PV isolation of non-isolated veins and mapping and ablation of all clinical and induced atrial tachycardias and flutters. These patients underwent an average of 1.4 + 0.55 catheter ablations. The CS patients underwent fewer repeat ablations than the NCS (1.1 + 0.3 vs 1.52 + 0.59, *P* = 0.040). There were no major complications from any of the catheter-based radiofrequency ablations.

### Follow-up

The average follow-up following catheter ablation was 2.99 + 2.35 years. This was 2.1 + 1.6 years for the CS patients and 3.4 + 2.5 years for the NCS patients. Six of 10 (60%) CS patients remained free of all atrial arrhythmias at the time of follow-up whereas 13 of 25 NCS patients (52%) remained free of all atrial arrhythmias at the time of follow-up (*P* = 0.723). Two additional NCS patients were free of atrial arrhythmias on antiarrhythmic therapy. Two patients died during follow-up of causes not related to their arrhythmia.

## Discussion

The main finding of this study is that among patients undergoing a radiofrequency catheter ablation for failed surgical maze, the CS maze technique of a classic Cox maze III operation isolated nearly all the PVs whereas other modalities of NCS procedures never isolated all the PVs. In addition, the most common clinical failure rhythm for the NCS procedures was AF and for the CS procedures was atrial flutter.

There have been no randomized trials comparing a CS to a NCS procedure for AF. However, there have been reports comparing populations of patients undergoing various surgical techniques for the treatment of AF. Gaynor et al. [11] compared a series of 276 patients who had undergone various iterations of the Cox maze procedure. This study had an extraordinarily long follow-up time for earlier versions of the Cox maze procedure. With 19.5% of patients on antiarrhythmic drugs, the original Cox maze I procedure had a 10-year freedom from AF rate of 75.4%, the Cox II procedure had a 10-year 83.6% freedom from AF rate, and the Cox maze III had a 10-year 89.3% freedom from AF. Follow-up was short for the Cox maze IV procedure which included many radiofrequency lesions. However, the 1-year freedom from AF, including those on antiarrhythmic drugs, was only 89% suggesting that it was going to be inferior to the Cox maze III procedure which had a similar freedom from AF after 10 years. In that study, the predictors of long-term success were the duration of AF prior to the surgery and the type of Cox maze procedure done. McCarthy et al. [6] examined 400 patients undergoing surgical treatment of AF. They compared 5 types of surgical AF procedures. At last follow-up, freedom from both AF and the need for a subsequent ablation was 90% for patients undergoing a classic maze procedure, 43% for a high intensity-focused ultrasound procedure, 79% for a LA maze procedure, 79% for a biatrial maze procedure, and 69% for PV isolation alone. For those patients who had only a LA maze procedure or PV isolation, the RA was the source for failure in 75%.

The best comparison of CS to NCS was reported by Stulak et al. [12]. They reported a matched control series of patients undergoing traditional CS Cox maze procedure compared to patients undergoing an identical operation but only using bipolar RF energy. Patients were matched for gender, age, New York Heart Association Class, AF type, and concomitant mitral valve surgery. They found that the CS maze was superior in all regards to the radiofrequency version of the same operation. At hospital discharge, 88% of patients with the CS procedure were in sinus rhythm compared to 63% for the radiofrequency maze. At 12 months of follow-up, 91% of the CS patients were free of AF versus only 76% for the radiofrequency group and at 15 months, the numbers were 91% and 61%. In addition, new permanent pacemaker implantation occurred in 25% of the radiofrequency group compared to only 5% of the CS group (*P* = 0.004). Antiarrhythmic medication at last follow-up was required in 75% of the radiofrequency ablation group as opposed to only 25% of the CS group (*P* < 0.05). Eighty-five of the patients in the radiofrequency group were still receiving warfarin anticoagulation at last follow-up compared to only 25% of patients in the CS group (*P* < 0.05). These authors concluded that “because transmurality can be assured, the standard CS Cox maze procedure remains the gold standard for the surgical treatment of AF.” The only logical explanation for the high failure rate for pulmonary vein isolation in our NCS group is that they did not have transmural lesions. One would anticipate that the surgeons would have a better opportunity to create transmural lesions in the operating room than ablationists can do in the EP lab with a catheter. However, when a through and through cut-and-sew incision is not made in the operations, the NCS lesions can be subject to the same limitations of performing it with a catheter in the electrophysiology laboratory: namely that as the tissue heals, conduction can recur. It is well known that the thickness of the atrium varies considerably, and radiofrequency, microwave, or other energies applied across the atrium might result in significant gaps in the lines the surgeons are attempting to create. Even electrophysiological mapping to document vein isolation at the time of surgery cannot prevent reconduction as healing occurs.

The study of Gillinov et al. [13] does suggest that doing a NCS operation is better than doing no AF operation at all. That study randomized patients undergoing mitral valve surgery with persistent or long-standing AF to receive no AF surgery, a pulmonary vein isolation ablation alone, or a pulmonary vein isolation plus a biatrial ablation. The surgical ablations were done using bipolar or unipolar RF. At 12 months 29.4% of patients receiving no AF ablation were in sinus rhythm and 63.2% of patients receiving AF ablation were in sinus rhythm. There was no difference between the two ablation strategies. Patients undergoing ablation had a 21.5% need for pacemaker implantation. The AF free outcomes in that study are remarkably similar to the study described above by Stulak et al. [12]. In that study, 76% of patients were free of AF and 25% required a pacemaker. One wonders if this study by Gillinov [13] might not have achieved the 91% freedom from AF reported by Stulak et al. [12] with only a 5% need for pacemaker had they used a CS instead of a NCS approach for patient’s randomized to AF surgery.

Despite the apparent superiority of a full CS maze procedure, it is our impression that relatively few surgeons still perform this operation. One reason may be the perceived difficulty of the procedure, but, in experienced hands, it can be done safely and efficiently. We have found that leaving the small 2-cm “button” of tissue near the LA appendage (which is subsequently cryoablated) greatly facilitates closure of the otherwise through and through box lesion around the PVs. Most centers seem to continue to perform the inferior alternatives to the standard Cox maze CS.

Many studies have examined the types of arrhythmias seen at catheter ablation following failed surgical maze procedures. None have directly compared the findings in patients undergoing CS to those undergoing a NCS maze procedures. In addition, many of the published studies are only reports of the patients with regular atrial tachycardias or flutters and do not include patients with AF recurring after surgery. Wazni et al. [14] examined 23 patients, all of whom underwent a classic CS maze procedure. Like our study, they found a high incidence of atrial tachycardias and flutters as the clinical failure rhythm. Sixty-five percent had atrial tachycardia or flutter, including 5 atrial tachycardias, 6 LA flutters, and 4 RA incisional flutters. In comparison to our study where we only had 10% with PV reconnection, they did have a 35% incidence of AF due to recovered PV conduction. At 1 year of follow-up, 82.6% were free of atrial arrhythmias off antiarrhythmic drugs after the ablation procedure. Magnano et al. [15] examined 20 patients who had only atrial tachycardia or flutter following a failed maze operation. They found 9 LA tachycardias, 6 typical and 1 atypical RA flutter, and 3 focal atrial tachycardias. They underwent a total of 26 electrophysiologic studies, and, at follow-up, 75% were in sinus rhythm. None of their patients underwent a CS maze, and a variety of energies including microwave, radiofrequency, cryoablation, and laser were used for the surgical maze. Since they excluded patients with AF as the failure rhythm after surgery, their findings could not be directly compared to those of our study. McElderry et al. [16] examined 22 patients who developed sustained atrial tachycardia after a surgical maze procedure. That study did not include any patients who received a pure CS procedure. The surgery was done using a combination of surgical incisions, radiofrequency ablation, and/or cryoablation. Half of the patients underwent a true CS box lesion around the veins and the other half had radiofrequency ablation. A total of 25 separate atrial tachycardias were mapped which included 15 in the RA and 10 the LA. Of the RA rhythms, 7 were isthmus-dependent atrial flutter, 7 non-isthmus-dependent flutters, and 1 focal atrial tachycardia. Seven LA tachycardias involved the roof and three involved the mitral annulus. All were ablated successfully, and they had no recurrences.

## Limitations

Our study has several limitations. The number of patients is small. None of the surgical maze procedures utilized electrophysiologic evaluation to document PV isolation or the integrity of the lines. The surgical procedures were done by many different surgeons at different institutions, and techniques may not have been fully standardized, especially for the NCS procedures. Our knowledge about the precise ablation protocol utilized for the NCS ablations was limited to what was in the operative notes and often did not include the number of applications or duration of ablation. However, they represent a “real-world” picture of what is being done by cardiac surgeons. Our findings could not be extrapolated to all patients undergoing a surgical maze as we only examined those patients referred for radiofrequency ablation after a failed surgical procedure.

## Conclusions

After a failed maze operation, the CS surgical procedure isolated nearly all PVs and the NCS procedure never isolated all PVs. The clinical failure rhythm was more frequently AF for NCS and AFl for CS. If the primary goal of the surgical procedure is to eliminate AF by PV isolation, then the CS procedure would appear to be the “gold standard.” More careful surgical attention to the mitral and tricuspid isthmus lesion sets would probably reduce many of the late surgical failures which are commonly atrial flutters involving these anatomical areas.
